# Do non-coordinating polymers function as host materials for solid polymer electrolytes? The case of PVdF-HFP

**DOI:** 10.1039/d3ta01853a

**Published:** 2023-06-26

**Authors:** Guiomar Hernández, Tian Khoon Lee, Máté Erdélyi, Daniel Brandell, Jonas Mindemark

**Affiliations:** a Department of Chemistry – Ångström Laboratory, Uppsala University Box 538 SE-751 21 Uppsala Sweden Guiomar.Hernandez@kemi.uu.se Jonas.Mindemark@kemi.uu.se; b Department of Chemical Sciences, Faculty of Science and Technology, Universiti Kebangsaan Malaysia 43000 UKM Bangi Selangor Malaysia; c Department of Chemistry – BMC, Uppsala University Box 756 75123 Uppsala Sweden

## Abstract

In the search for novel solid polymer electrolytes (SPEs), primarily targeting battery applications, a range of different polymers is currently being explored. In this context, the non-coordinating poly(vinylidene fluoride-*co*-hexafluoropropylene) (PVdF-HFP) polymer is a frequently utilized system. Considering that PVdF-HFP should be a poor solvent for cation salts, it is counterintuitive that this is a functional host material for SPEs. Here, we do an in-depth study of the salt dissolution properties and ionic conductivity of PVdF-HFP-based electrolytes, using two different fabrication methods and also employing a low-molecular-weight solvent analogue. It is seen that PVdF-HFP is remarkably poor as an SPE host, despite its comparatively high dielectric constant, and that the salt dissolution properties instead are controlled by fluorophilic interactions of the anion with the polymer.

## Abstract 2

PVdF-HFP does not coordinate to lithium ions, therefore it is a poor polymer host for solvent-free polymer electrolytes.

## Graphical abstract

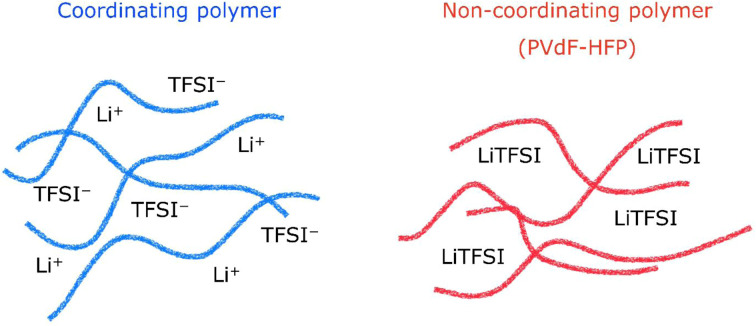

## Introduction

The current renaissance for polymer-based solid-state batteries has led to a boom in the exploration of different types of host materials that can contain useful salts – primarily Li-salts – rendering solid polymer electrolytes (SPEs).^1,2^ In this context, the fundamental theories on ionic transport in these materials which put strict requirements on the types of polymer that can function in SPE systems, are sometimes overlooked. If the limited subcategory of SPE materials that possess “structural diffusion” (where ion hopping occurs between fixed sites in the matrix, analogous to ceramic ion conductors)^3,4^ is excluded, the ionic transport needs to be correlated to the segmental mobility of the macromolecule, in a mechanism that comprises sequential exchange of the ligands.^5,6^ The archetypical example is poly(ethylene oxide) (PEO), that dissolves Li salts particularly well, and where a percolating movement of the macromolecular solvent is correlated to the transport of ions, well in accordance with free volume theory.^7^ Thereby, cation-polymer coordination is a prerequisite for a well-functioning SPE.

These fundamental theories on ionic transport in SPEs, that date back to the 1980s,^5,8^ tell a story of primarily two polymer properties being necessary for ionic conduction: the polymer needs to be flexible enough to provide the segmental motion necessary to transport ions, and the polymer matrix must dissolve the salt to a necessary degree in order to have ions to conduct in the first place. The first renders ionic mobility, and the second controls the amount of charge carriers: the conductivity is the product of these properties. This essentially means that the *T*_g_ of the SPE system needs to be lower than the operating temperature for the electrolyte, and thermodynamically that the total binding energy of the polymer–ion interactions is higher than the combined lattice enthalpy of the salt and polymer–polymer interactions of the host material. Regarding the latter, it has been made clear that the donor number is in fact considerably more of a key property than the dielectric constant of the macromolecular medium.^9^ While a strong dielectric can shield the charges between the ionic species and prevent pairing and clustering, it is not a strong indicator of the necessary ion–polymer interaction. However, the donor number is.

Poly(vinylidene fluoride) (PVdF) and the closely related poly(vinylidene fluoride-*co*-hexafluoropropylene) (PVdF-HFP) can perhaps at first glance constitute appropriate host materials for SPEs: the *T*_g_ is fairly low (around −35 °C), and the dielectric properties are appealing for a polymeric material. It is also a good electronic insulator and possesses a wide electrochemical stability window, which are necessary properties for battery applications.^10,11^ On the other hand, these compounds are highly crystalline, especially PVdF but also PVdF-HFP, and the crystalline phases cannot themselves host any ions. Moreover – and the key point here – is their low donor number and poor complexation of salt cations, not least Li^+^.

It should be noted that in most electrolyte studies using PVdF or PVdF-HFP, these polymers constitute either a basis for a gel comprising also a liquid phase, or are heavily plasticized with low-molecular-weight components.^12,13^ While these approaches can generate fairly well-performing electrolytes in terms of conductivity, they often suffer from the same problems as liquid electrolyte systems in terms of electrochemical stability, long-term chemical stability and safety, directly correlated to the amount and nature of the liquid component. Since the transport processes in these materials are less related to the polymeric component – but instead to the liquid phase or components – it is doubtful whether they should be considered ‘polymer electrolytes’, since the polymer is more of a passive container for the liquid phase than part of the conductive medium. This is often seen in the conductivity behavior, which for gels and liquids typically does not show the pronounced Vogel–Fulcher–Tammann-type behavior seen for SPEs.^14^ There also exists recent examples of using high salt concentrations in PVdF, within the so-called polymer-in-salt-electrolyte (PISE) domain.^15,16^ Here, however, the ions are rather conducted through hopping between different clusters in a Grotthuss-type mechanism, and the polymer is again a rather passive component.^17^ Nevertheless, PVdF and PVdF-HFP are often promoted as good polymer hosts for polymer electrolytes,^11,16,18,19^ and are as such described in the same context as PEO, polycarbonates, polyesters, *etc.*; polymers that have fundamentally different ion coordinating properties.

In this study, we explore the limits of the true SPE properties of PVdF and PVdF-HFP analogues, by focusing on their coordination chemistry and conduction properties. We show that the fabrication technique is critical for the electrolyte performance, which suggests that solvent residues might be crucial for some of the more positive results reported previously in literature.

## Experimental section

### Materials

Lithium bis(trifluoromethanesulfonyl)imide salt (LiTFSI; BASF), poly(vinylidene fluoride)-*co*-hexafluoropropylene (PVdF-HFP; Kynar Flex 2801 Arkema), 1,1,1,3,3,5,5,5-octafluoropentane (octafluoropentane, Fluorochem), acetone (Sigma-Aldrich, 99.8%, anhydrous), acetonitrile (ACN; Sigma-Aldrich, 99.8%, anhydrous).

### Preparation of polymer electrolyte films by solvent casting

PVdF-HFP and LiTFSI (5 and 15 wt%) were dissolved in acetone with a ratio of polymer to solvent of 0.05 g ml^−1^. The polymer electrolyte solution (2 ml) was cast in PTFE molds and the solvent removed by heating under vacuum for 60 h. The sample preparation was done in an argon glovebox.

### Preparation of polymer electrolyte films by hot pressing

The two powders PVdF-HFP and LiTFSI (5 and 15 wt%) were mixed with a MM 400 mixer mill (Retsch). Afterwards, the powder was heated for 1 h at 140 °C and then hot pressed between two PTFE sheets at 20 MPa and 140 °C for 1 h. The sample preparation was done in an argon glovebox.

### Ionic conductivity measurements

The ionic conductivity of the polymer electrolytes was measured using electrochemical impedance spectroscopy (EIS) with an SI 1260 Impedance Gain-Phase analyzer (Schlumberger) at a frequency range of 1 Hz to 10 MHz with an amplitude of 10 mV between 30 and 100 °C. The polymer electrolyte films were cut in discs of 10 mm diameter corresponding to 0.785 cm^2^ and sandwiched between stainless steel electrodes in coin cells. The cells were annealed at 140 °C for 1 h to improve the interfacial contact between the electrolyte and electrodes and then cooled down to room temperature. The samples were equilibrated at each temperature for 20 min before measurement. The data was analyzed using ZView software (Scribner Associates) employing a modified Debye equivalent circuit.^20^ The ionic conductivity, *σ*, was calculated from the equation *σ* = *t*/(*R* × *A*), where *t* is the polymer electrolyte thickness, *R* is the bulk resistance and *A* is the area of the electrolyte film in contact with the blocking electrodes.

### Thermogravimetric analysis (TGA)

Solvent residues and degradation temperatures were analyzed by TGA on a TA5500. The samples were exposed to ambient air for a few minutes during the transfer from the glovebox to the instrument. The measurements were carried out by heating the samples from room temperature to 500 °C at a heating rate of 10 °C min^−1^ under nitrogen atmosphere.

### Differential scanning calorimetry (DSC)

Thermal transitions in the polymer systems was analyzed by DSC on a Mettler Toledo DSC 3+. The samples were hermetically sealed in aluminum pans in an argon-filled glovebox. Two cooling/heating cycles were carried out between −60 and 200 °C with a heating ramping speed of 10 °C min^−1^ under nitrogen. The second heating ramp was used for measurement and plotting.

### Fourier transform infrared spectroscopy (FTIR)

FTIR spectra were acquired on a Bruker Vertex 70v FT-IR spectrometer with a RT-DLaTGS detector in ATR mode with a Heated Diamond Crystal ATR Puck (Specac). The measurements were performed in the wavenumber range 5000–400 cm^−1^ with 32 scans and a resolution of 4 cm^−1^. The samples were exposed to air for a few minutes while transferring them from the glovebox to the equipment and during the measurement.

### X-ray diffraction (XRD)

Solubility of LiTFSI in PVdF-HFP was analyzed by XRD on a Bruker D8 Advance diffractometer with Cu K_α_ radiation equipped with a LynxEye XE-T detector in Bragg–Brentano geometry. The polymer electrolyte films prepared through casting or hot pressing with 15 wt% LiTFSI were compared to the pristine salt and a film of PVdF-HFP without salt. The latter was prepared by hot pressing the powder between Teflon sheets for 1 h at 140 °C. The samples were exposed to ambient air during these experiments.

### Nuclear magnetic resonance (NMR) spectroscopy

Quantification of the solubility of LiTFSI in the model fluorinated liquid 1,1,1,3,3,5,5,5-octafluoropentane was done through ^7^Li NMR. The experiments were performed on a 400 MHz JEOL ECZ 400S NMR spectrometer at 25 °C for 16 scans and 10 s relaxation delay. The reference solution was 1 mmol kg^−1^ LiCl in D_2_O. The concentration of the samples used for calibration were 1, 5 and 10 mmol kg^−1^ LiTFSI in acetonitrile. They were placed in an NMR insert (Wilmad) with the reference sample in the outer tube. To quantify the solubility of LiTFSI in the octafluoropentane, a saturated solution of LiTFSI in the solvent was prepared. It was heated for 2 h at 40 °C and at room temperature for 3 days. Afterwards, the solution was centrifuged at 7000 rpm for 5 min and the supernatant taken for NMR analysis.

Chemical shift perturbation (^19^F, ^7^Li), HOESY (^7^Li, ^19^F) and DOSY (^7^Li, ^19^F) experiments were performed on an Agilent 400 MR DD2 spectrometer equipped with a OneNMRProbe at 25 °C. The chemical shift was referenced to DMSO-d_6_, added as an external reference in a capillary to the 5 mm NMR tube. ^19^F NMR spectra were acquired with 8 scans, 0.7 s D1 delay, 16 384 acquired points and 43 103 Hz spectral window. ^7^Li NMR spectra were acquired with 4 scans, 1 s D1 delay, 16 384 points and 1923 Hz spectral window. DOSY spectra were run using the DgcsteSL_cc (Gradient Compensated Stimulated Echo with Spin-Lock and Convection Compensation) pulse sequence with 16 scans and 1.5 s D1 delay, and using an array of 25 gradients between 82 and 2047. ^19^F,^19^F NOESY^21,22^ and ^19^F,^7^Li HOESY^23^ were acquired with *t*_mix_ 700–1000 ms, D1 2 s, and 8 scans.

## Results and discussion

The method used to prepare solid polymer electrolytes can affect the properties of the material, especially if solvent casting is used and solvent residues remain in the sample. This relation was investigated with PVdF-HFP and LiTFSI (5 and 15 wt%) prepared through solvent casting and hot press methods. The solvent used for solvent casting was acetone because it has a lower boiling point and easier removal than other commonly used solvents such as DMSO and DMF.^24^

**Fig. 1:**
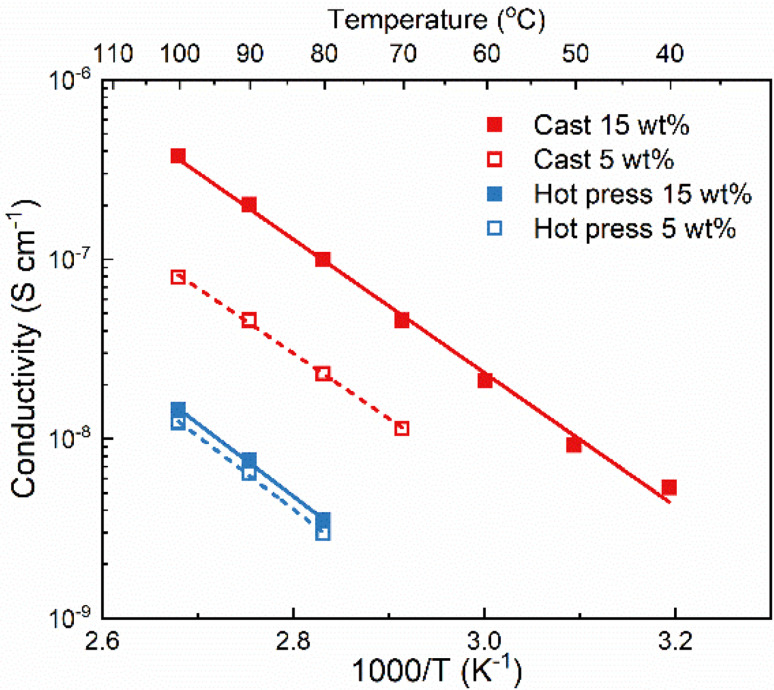
Ionic conductivity as a function of temperature of PVdF-HFP with 5 and 15 wt% LiTFSI (empty/dash and filled/straight symbol/line, respectively) prepared with solvent-cast (red) and hot-pressed (blue) methods.

The ionic conductivity of the films was measured with electrochemical impedance spectroscopy at different temperatures and the results are shown in Fig. 1. The samples from solvent casting presented higher ionic conductivity than the samples from hot pressing. For example, for 5 wt% LiTFSI the ionic conductivity was 8 × 10^−8^ S cm^−1^ for solvent cast and 1.2 × 10^−8^ S cm^−1^ for hot-pressed samples at 100 °C; *i.e.*, almost an order of magnitude difference. Similar differences have also been reported for other polymer electrolyte systems, for example polyvinylalcohol (PVOH) with lithium triflate (LiCF_3_SO_3_).^25^ Furthermore, both samples show an Arrhenius-type behavior of the conductivity (*i.e.*, a straight line in the log *σ vs.* 1/*T* plot), indicating that the ion transport is not strongly coupled to the segmental motion of the polymer host.

Comparing the effect of the salt concentration on both samples, a more pronounced increase in ionic conductivity was observed for the solvent-cast sample as compared to the hot-pressed sample when increasing the salt content from 5 to 15 wt%. This behavior is likely due to the interaction between the salt and the casting solvent, that prevents complete evaporation of the solvent. Therefore, increasing the salt concentration using the solvent casting method could lead to higher amount of solvent residues and, consequently, increasing the ionic conductivity.^24^ This effect is not seen for the hot-pressed sample where the increase in ionic conductivity is rather limited. This could be due to the low solubility of LiTFSI in PVdF-HFP, which might already be saturated at 5 wt% salt.

Regardless of the sample preparation protocol, the ionic conductivity is extremely low (3.8 × 10^−7^ and 1.4 × 10^−8^ S cm^−1^ for both solvent-cast and hot-pressed samples with 15 wt%, respectively) even at high temperatures (100 °C), indicating that PVdF-HFP is not a good matrix for truly solid polymer electrolytes. The lack of functional groups able to coordinate to the salt results in poor solvation ability of the polymer which is compensated by the solvent residues in the cast sample. Higher ionic conductivity might seem beneficial and the presence of solvent residues could improve the wettability and the formation of the solid electrolyte interphase. However, solvent residues can also act as impurities leading to battery problems, such as side reactions lowering the coulombic efficiency.^2^ Regardless of their impact on the cell performance, solvent residues will definitely affect the solubility of the salt, and the transport mechanism which will thereby no longer be the same as in a truly solid system.

**Fig. 2:**
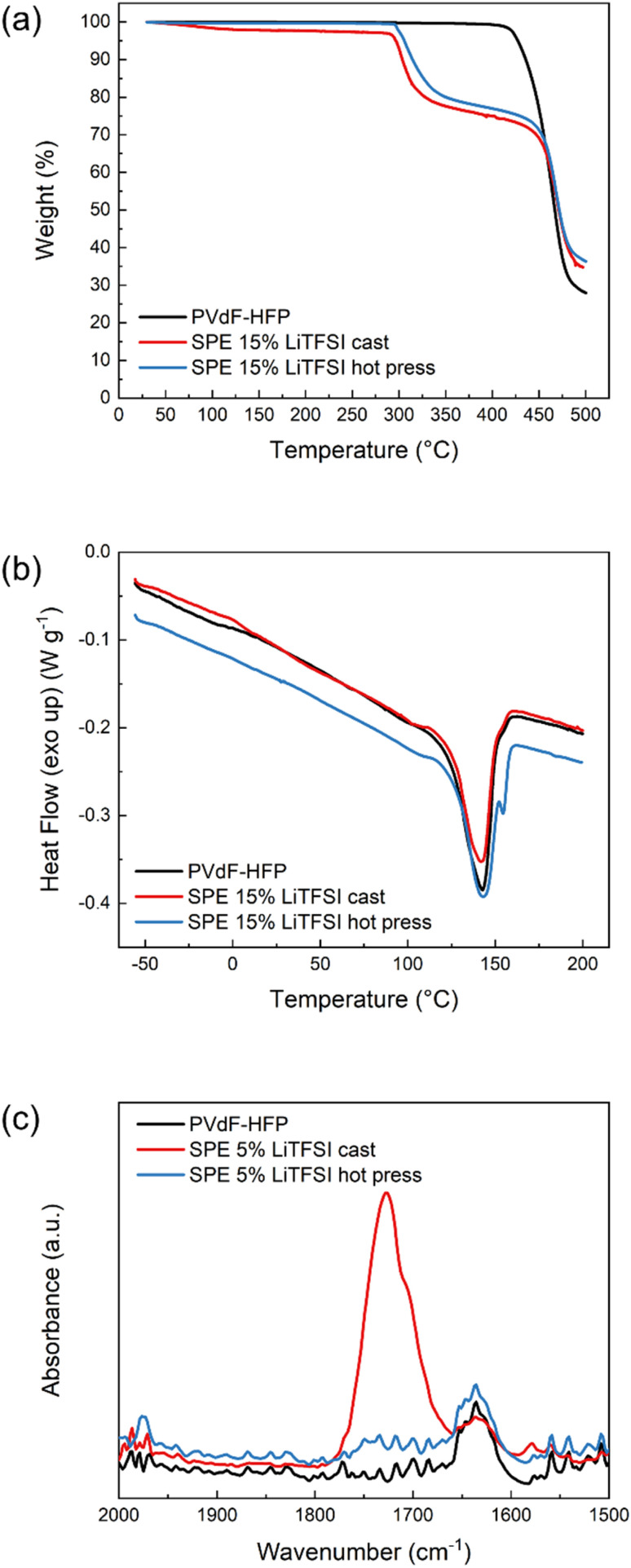
(a) Thermogravimetric analysis, (b) differential scanning calorimetry (DSC) and (c) FTIR absorption spectra in the carbonyl region of PVdF-HFP (black), SPE cast (red) and SPE hot press (blue).

The presence of solvent residues is further confirmed with TGA. As can be seen in Fig. 2a, the solvent-cast sample has an initial mass loss of around 3% before 200 °C which is not present in the pure polymer or the hot-pressed SPE. Both SPE samples show decomposition of LiTFSI at around 300 °C and polymer host degradation at 450 °C, similar to pure PVdF-HFP. To further investigate the thermal properties of these materials, DSC was performed and the results shown in Fig. 2b. None of the samples show a clear *T*_g_ which could be due to their high crystallinity. The pure PVdF-HFP features a melting peak at 143 °C which is still present in the SPEs, indicating that the salt does not interfere much with the crystallinity of the polymer host. The hot-pressed SPE furthermore exhibits an additional sharp peak above 150 °C which could be attributed to the solid–solid transition of LiTFSI,^26^ suggesting there is undissolved salt in the hot-pressed SPE. In the case of the solvent-cast SPE, the area of the melting peak is smaller than the pristine polymer, suggesting that the presence of LiTFSI–acetone clusters decreases the degree of crystallinity. However, in the hot-pressed sample, the DSC reveals the poor solubility of LiTFSI in PVdF-HFP which explains the low conductivity of the sample, regardless of the salt concentration. In addition, FTIR was performed on these samples to further investigate the presence of solvent residues. As can be seen in Fig. 2c, the solvent-cast sample shows an absorption band at 1726 cm^−1^ corresponding to the carbonyl stretching in acetone. Another peak is observed at slightly lower wavenumbers which has been previously attributed to lithium ions coordinated by acetone.^27^ These results confirm that solvent residues are still present in the cast sample, solubilizing the LiTFSI and enhancing the ionic conductivity. Therefore, the method used to prepare SPEs has a critical influence on the final properties of the material.

**Fig. 3:**
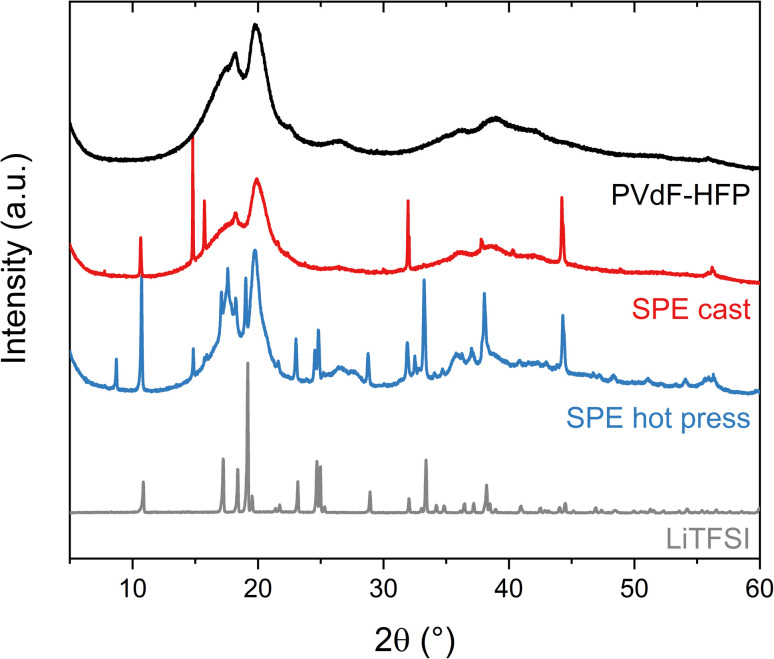
X-ray diffraction (XRD) patterns of the LiTFSI powder (black), PVdF-HFP film (gray), SPE cast (red) and SPE hot press (blue) with 15 wt% LiTFSI in PVdF-HFP.

The ability of the polymer matrix to solvate and therefore dissolve LiTFSI was further examined with XRD (Fig. 3). These experiments were performed on the pure lithium salt and PVdF-HFP film, as well as the solvent-cast and hot-pressed samples of 15 wt% LiTFSI in PVdF-HFP. The polymer film without salt shows two broad peaks around 20° and 18°, revealing the formation of the semi-crystalline *α*-phase of PVdF.^28^ The sample prepared with the hot press method shows broad peaks corresponding to the PVdF, similar to the polymer film without salt, and sharp peaks that reveal the presence of crystalline salt. The slight changes in the intensities and peak position of the pattern compared to the pure salt could be due to the thermal history of the sample which could favor the recrystallization of the salt in a different crystal structure and preferred orientation.^29,30^ Nevertheless, this indicates that the solubility of LiTFSI in PVdF-HFP is very low and that its plasticizing effect is limited, since the peaks from the polymer have not changed significantly. However, in the case of the solvent-cast sample, the intensity of the broad peak at 18° from PVdF has diminished and fewer sharp peaks appear in the diffractogram. This means that LiTFSI or LiTFSI–acetone complexes are partly solubilized in the PVdF-HFP matrix, thereby acting as plasticizers which hinder the crystallization of PVdF. Overall, the XRD results indicate a low solubility of LiTFSI in PVdF-HFP, especially if no other solvent is present.

**Fig. 4:**
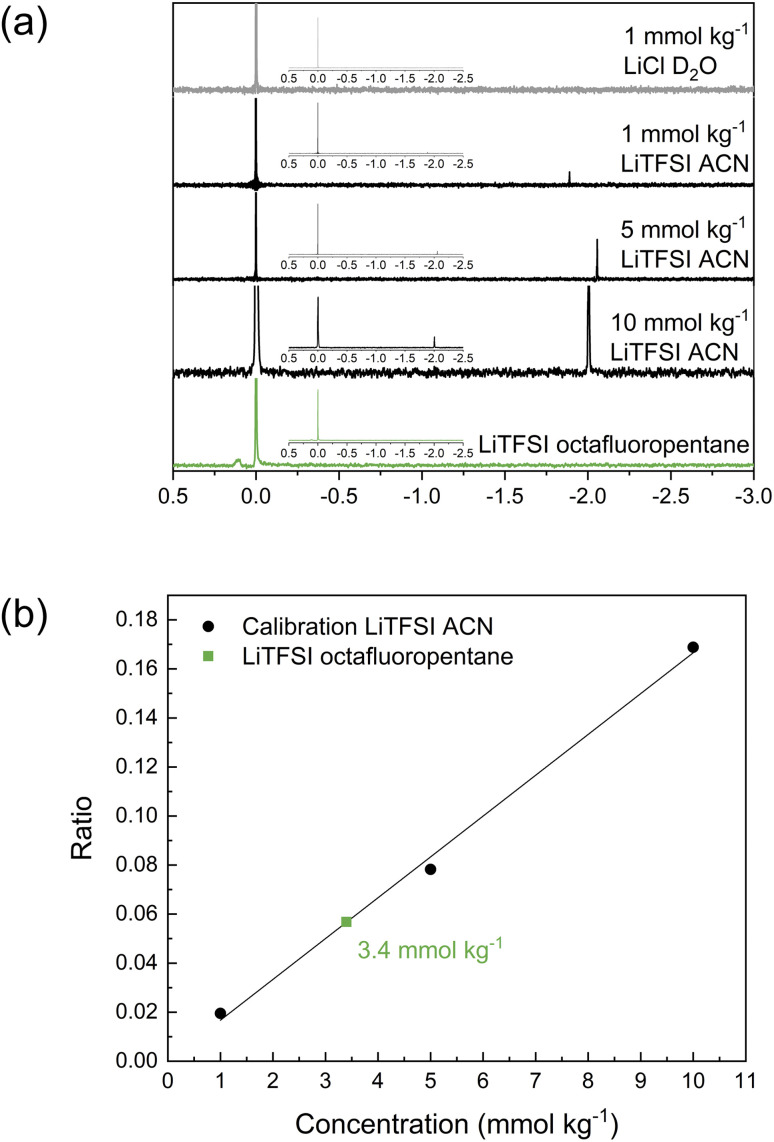
(a) ^7^Li NMR spectra of the reference LiCl in D_2_O, LiTFSI in acetonitrile 1, 5 and 10 mmol kg^−1^ and LiTFSI in octafluoropentane, inset shows the full spectra. (b) Calibration curve from the ratio of the integral of the samples to the reference including the ratio and concentration of LiTFSI in octafluoropentane.

As the investigations thus far have indicated limited solubility of the salt in the highly fluorinated PVdF-HFP matrix, the question arises of what the maximum solubility of LiTFSI really is in this type of polymer. One technique to investigate the solubility that also highlights the interaction between LiTFSI and fluorinated hydrocarbons is ^7^Li NMR. For these experiments, octafluoropentane was used as a liquid-phase model system to represent PVdF-HFP. The reference solution was LiCl in D_2_O, and different concentrations of LiTFSI in acetonitrile (1, 5 and 10 mmol kg^−1^) were used for calibration. The obtained spectra in Fig. 4a show the two peaks corresponding to LiCl at 0 ppm and LiTFSI in acetonitrile at *ca.* −2 ppm. The relative area between these two peaks changes with the concentration of the sample. The ratio of the integral of the sample to the reference was used for the calibration plot (Fig. 4b) to quantify the concentration of LiTFSI in octafluoropentane based on the obtained ratio. A saturated solution of LiTFSI in octafluoropentane was prepared by heating and stirring, followed by a centrifugation step to collect the supernatant for ^7^Li NMR analysis. This sample features a small peak at 0.1 ppm indicating that the coordination structure and environment of Li^+^ in octafluoropentane is different to acetonitrile. The highly fluorinated solvent shifts the signal to a positive value. The ratio of the integral between the sample and reference corresponds to a concentration of 3.4 mmol kg^−1^ or 0.1 wt% LiTFSI. These experiments indicate the extremely low solubility of LiTFSI in octafluoropentane, and therefore further confirms the poor ability of fluorinated hydrocarbons to solvate and coordinate to lithium ions and/or TFSI anions.

**Fig. 5:**
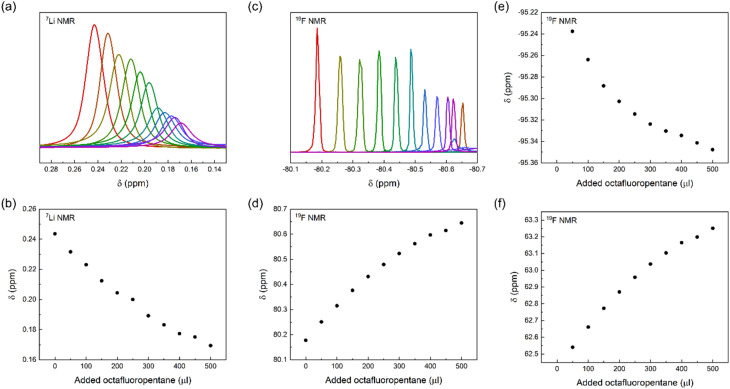
(a) Chemical shift perturbation experiments by addition of 50 μl aliquots of octafluoropentane (FL) into a diethylamine solution of LiTFSI using ^7^Li and ^19^F NMR. Here, (a) and (b) show the ^7^Li NMR chemical shift change, (c) and (d) the ^19^F NMR chemical shift change detected on TFSI, whereas (e) and (f) the ^19^F NMR chemical shift change detected on octafluoropentane during the titration.

To gain further insight into the interaction of LiTFSI and a fluorinated liquid, we titrated LiTFSI dissolved in trimethylamine with octafluoropentane in 50 μl aliquots, 0 to 500 μl, using parallel ^19^F and ^7^Li NMR detection of chemical shift perturbations. We observed chemical shift perturbations, shown in Fig. 5, that may be interpreted either as a consequence of the interaction of TFSI and Li^+^ with octafluoropentane, or by the alteration of the ionic strength of the solution upon stepwise addition of a less polar co-solvent to the LiTFSI solution. As the observed chemical shift changes were deemed inconclusive, we attempted the detection of Overhauser effects, which could further prove the close association of the constituents of a LiTFSI with octafluoropentane solution, and hence acquired ^19^F,^19^F NOESY and ^19^F,^7^Li HOESY spectra. The lack of NOE between TFSI and octafluoropentane and the absence of HOE between Li^+^ and octafluoropentane may be due to the absence of direct interaction between the constituents, or due to the too low concentration of LiTFSI of the sample preventing the detection of very weak intensity cross-peaks. By acquiring DOESY spectra for a solution of LiTFSI in octafluoropentane, (*D*_Li_ = 5.8 ± 0.26 ×10^−10^ m^2^ s^−1^; *D*_TFSI_ = 5.99 ± 0.12 ×10^−10^ m^2^ s^−1^; *D*_FL_ = 11.61 ± 0.06 ×10^−10^ m^2^ s^−1^) strong interaction and hence co-diffusion of Li^+^ and TFSI could be observed, but no specific interaction of Li^+^ and TFSI with octafluoropentane. As a control experiment, we measured the translational diffusion coefficients of the components of LiTFSI dissolved in diethylamine (*D*_Li_ = 11.34 ± 0.07 ×10^−10^ m^2^ s^−1^; *D*_TFSI_ = 11.27 ± 0.05 ×10^−10^ m^2^ s^−1^; *D*_Et2N_ = 33.34 ± 0.03 ×10^−10^ m^2^ s^−1^), which confirmed the co-diffusion of Li^+^ and TFSI also in a polar environment, whereas this measurement indicated no direct interaction of these ions with diethylamine. The latter observation further confirms that the diethylamine, which was used as a co-solvent to increase LiTFSI solubility in the chemical shift perturbation and NOESY/HOESY experiments, does not interfere with the LiTFSI-octafluoropentane interaction. Our conclusion from these NMR experiments is that there is no direct interaction between Li^+^ or TFSI with fluorinated solvents, such as octafluoropentane, instead TFSI as counterion increases Li^+^ solubility. On an atomic level, a fluorine–fluorine interaction^31–34^ of TFSI and octafluoropentane – fluorophilicity – is the likely intermolecular force that enables solubilization of the salt in the non-polar fluorous solvent environment. To further confirm this effect of the anion, we carried out control experiments with LiCl. The results showed that LiCl is not soluble in octafluoropentane as no signal was detected using ^7^Li NMR. These results indicate that the little solubility of LiTFSI in octafluoropentane originates from the anion–solvent fluorophilicity and not from the coordination of the fluorous solvent to the lithium cation.

## Conclusions

The poor solvating ability of PVdF-HFP with LiTFSI and the significant impact of the fabrication method of solid polymer electrolytes has been confirmed with a combination of employed techniques. The higher ionic conductivity observed for the cast sample, compared to the hot-pressed sample, is due to the remaining solvent residues, confirmed with TGA. Undissolved salt remains in the hot-pressed sample, as seen with DSC and XRD, confirming the low solubility of LiTFSI in PVdF-HFP. Regardless of the manufacturing method, both electrolyte systems showed low ionic conductivity, and the conductivity observed is highly dependent on solvent residues. These results corroborate the poor ability of PVdF-HFP to act as a solid polymer electrolyte.

To quantify the solubility of the salt and investigate the salt–solvent interactions, a liquid-phase model system with octafluoropentane as solvent was used. Quantitative ^7^Li NMR gave a concentration of 0.1 wt% LiTFSI in the fluorinated solvent, confirming the low solubility of LiTFSI in a fluorous solvent environment. Finally, it has been shown that the fluorophilicity of a highly fluorinated anion (such as TFSI) and solvent (octafluoropentane which could be correlated to PVdF-HFP) is what promotes a slight solubility of Li^+^ and not the ability of the fluorinated matrix to coordinate to the cation.

Overall, these results confirm that PVdF-HFP is not a good host for solvent-free polymer electrolytes. Not only because of the low solubility of the salt in the polymer matrix, but also the low ionic conductivity of the system. Consequently, this polymer should not be reported as solid polymer electrolyte host because it does not have the requirements needed to be treated as such.

## Author contributions

DB and JM devised the idea for the project. GH, TKL and ME performed the experiments, GH wrote the original draft and all authors contributed to the planning, analyzing, reviewing and editing of the manuscript.

## Conflicts of interest

There are no conflicts to declare.

## Supplementary Material
